# The Use of an Alternative Extraoral Periapical Technique for Patients with Severe Gag Reflex

**DOI:** 10.1155/2016/3206845

**Published:** 2016-07-31

**Authors:** Mauro Henrique Chagas e Silva, Marcelo Santos Coelho, Mariane Floriano Lopes Santos, Carolina Oliveira de Lima, Celso Neiva Campos

**Affiliations:** ^1^Private Practice, Rua Professor Alberto Pacheco 125/107, 36570-000 Viçosa, MG, Brazil; ^2^Endodontic Department, Faculdade de Odontologia São Leopoldo Mandic, Rua Emilo Ribas 776/13, 13025-141 Campinas, SP, Brazil; ^3^Department of Clinical Dentistry, Universidade Federal de Juiz de Fora, Rua José Lourenço Kelmer, S/N, 36036-900 Juiz de Fora, MG, Brazil

## Abstract

Gag reflex is a physiologic mechanism that promotes contraction of the muscles of the tongue and pharyngeal walls. Different factors, including intraoral radiographic films and sensors, may trigger this reflex. Patients with severe gag reflex may not be able to tolerate the presence of intraoral radiographic films or sensors during root canal therapy (RCT). This factor may prevent an appropriate intraoral radiograph, which is important in RCT. Different approaches have been used to facilitate dental procedures in patients suffering from severe gag reflex. The use of an extraoral radiographic technique is an alternative method to obtain working length confirmation in patients with severe gag reflex. In this report of 2 cases, the use of an extraoral radiographic technique as an alternative approach during RCT in patients with severe gag reflex associated with phobic behavior and trismus was successfully demonstrated.

## 1. Introduction

Gag reflex is a natural response of a human body to eliminate foreign bodies from the pharynx, larynx, or trachea. Stimulation of the soft palate or the posterior third of the tongue may trigger this reflex resulting in contraction of the muscles of the tongue and pharyngeal walls. Although physiological, this reflex may prevent proper dental management and result in increased dental phobia [1, 2].

Periapical radiographs are important during root canal therapy (RCT) because they help the dental professionals to verify the appropriate working length (WL), gutta-percha points adjustment, and presence of voids in the root canal filling before completion of the treatment. Even after introduction of electronic apex locators (EALs), radiographs are important for WL determination [3]. However, patients with severe gag reflex are not able to tolerate intraoral films or sensors for intraoral radiography [4].

Fisher first reported on the use of an extraoral technique by using an occlusal film for third molar evaluation [5]. Newman et al. have described an alternative extraoral technique to achieve periapical radiographs in patients with trismus or severe gag reflex; this technique is suitable for both mandibular and maxillary molars [6]. This extraoral radiographic technique may be used as an appropriate alternative for satisfactory periapical radiographs in patients who cannot tolerate the conventional technique [7].

In the following report of 2 cases, the extraoral technique was successfully used during RCT in patients with severe gag reflex associated with trismus and phobic behavior.

## 2. Case Report Number 1

A 28-year-old male patient presented with a chief complaint of dull and intermittent pain in his maxillary right first molar. The patient presented with a previous periapical radiograph that was acquired in a radiology service unit; the patient conveyed that his gag reflex had made him vomit during the periapical radiography. After clinical examination, an extensively carious lesion was observed on the mesial and occlusal aspects of the tooth. The sensitivity test performed using a cold stimulus (Coltene, Vigodent, France) and the electrical pulp tests were positive, and no pain was present during the percussion and palpation tests. The diagnosis of irreversible pulpitis was confirmed. In addition to the gag reflex, the patient also had phobia of dental procedures.

The initial approach was by using the protocol proposed by Robinson et al. [8] to gain the patient's trust and to let him know the possibility of painless dental procedures. The superior posterior and middle alveolar nerves were blocked using 2% alphacaine with 1 : 100,000 epinephrine (DFL Ind. e Comércio, Rio de Janeiro, Brazil). Additionally, a small amount of anesthetic was infiltrated in the palatal aspect. During the first visit, an endodontic access cavity was prepared using the carbide bur 1557 (Microdont, São Paulo, Brazil) and Endo-Z (Dentsply, Rio de Janeiro, Brazil). Rubber dam placement was achieved after the endodontic access and pulpectomy was performed using hand k-files of sizes 08, 10, and 15. The gag reflex prevented further procedures and a temporary restoration (intermediate restorative material [IRM], Dentsply, Petrópolis, Brazil) was placed.

In the second visit, the rubber dam was placed and, in order to avoid an intraoral radiographic procedure, WL determination was achieved using the EAL Apit 5 (Osada, Tokyo, Japan). The root canal shaping was performed using Easy Logic 25.06 nickel-titanium (NiTi) files (Easy Dental, Belo Horizonte, Brazil) under copious irrigation with 5.25% sodium hypochlorite ([NaOCl] Fórmula e Ação, São Paulo, Brazil) and 17% ethylenediaminetetraacetic acid ([EDTA] Fórmula e Ação, São Paulo, Brazil).

**Figure 1:**
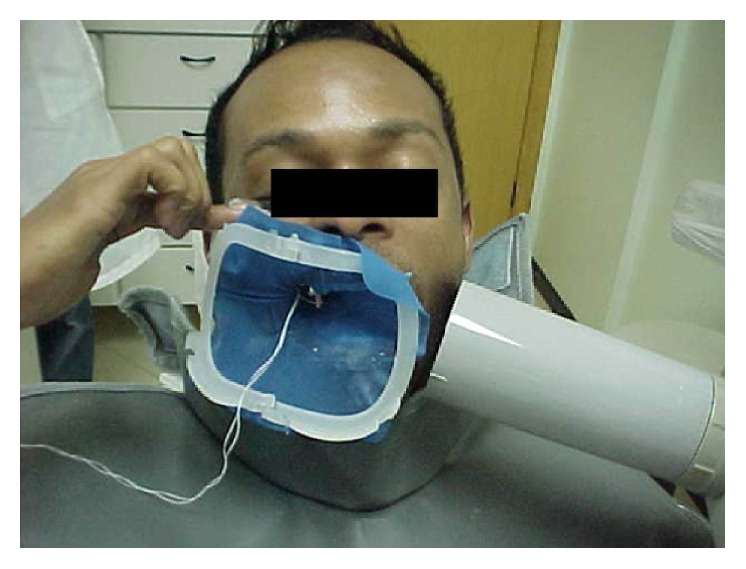
Extraoral technique used for maxillary molar in case report 1.

The extraoral technique was used to verify the appropriate lengths of the gutta-percha points (Odous, Belo Horizonte, Brazil). With the patient in sitting position with maximum mouth opening, the radiographic film Kodak E-Speed (Eastman Kodak Company, Rochester, USA) was positioned on the external aspect equivalent to the tooth position. The cone of the radiographic device was placed in the opposite direction in order to make the X-rays reach the bisection of the angle formed between the film and the tooth resulting in approximately −55° angle and perpendicular to the film (Figure 1). Due to the long object-film distance, the exposition time was increased to 1.5 s.

**Figure 2:**
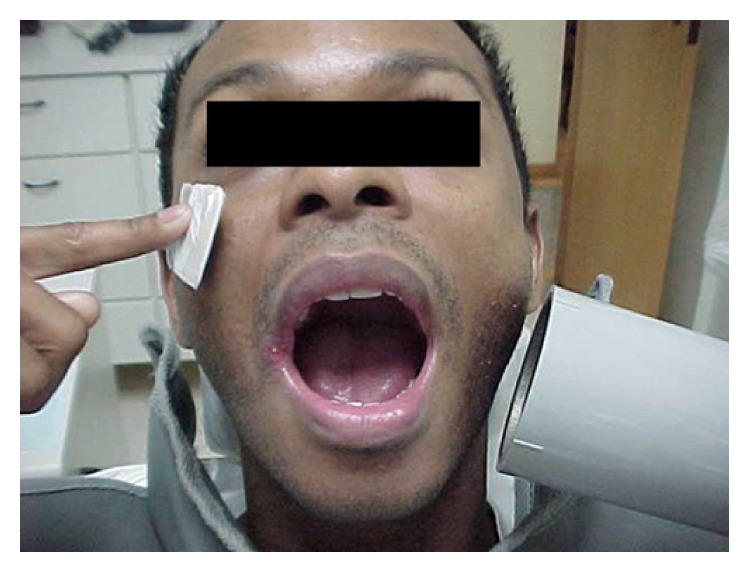
Extroral technique for final radiograph in root canal therapy of the maxillary molar.

**Figure 3:**
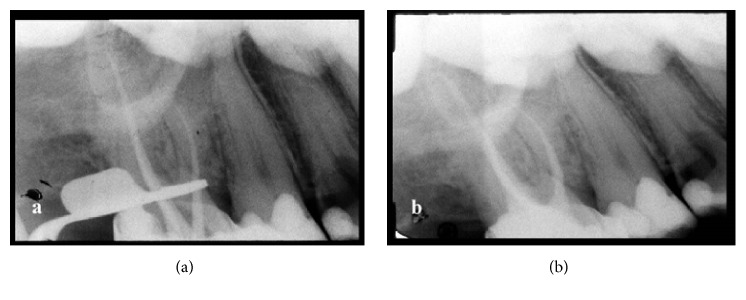
(a) Radiograph with the gutta-percha points: case report number 1. (b) Final radiograph: case report number 1.

After the radiographic evaluation of the WL with the gutta-percha points in position, the final root canal filling was performed using a warm vertical compaction with the endodontic sealer Endofill (Dentsply, Rio de Janeiro, Brazil) and gutta-percha (Odous de Deus, Belo Horizonte, Brazil). The temporary filling material used in the crown was IRM. Postoperative radiography was also performed using the extraoral technique (Figures 2 and 3).

## 3. Case Report Number 2

A 28-year-old female patient presented to the clinic with a chief complaint of pain in the mandibular left first molar. The cold and electric pulp tests were positive for the referred tooth, and there was no pain on percussion or palpation. The patient was diagnosed with irreversible pulpitis. The patient had brought a previous periapical radiograph performed in a radiology service unit.

An inferior alveolar nerve block was achieved using 3.6 mL of 2% lidocaine with 1 : 100,000 epinephrine (DFL Ind. e Comércio, Rio de Janeiro, Brazil) followed by buccal and lingual infiltration using 4% articaine with 1 : 100,000 epinephrine (DFL Ind. e Comércio, Rio de Janeiro, Brazil). In the first visit, the endodontic access cavity was prepared using the carbide bur 1557 and Endo-Z. After rubber dam placement, pulpectomy was performed using k-files of sizes 08, 10, and 15. In addition to the gag reflex, the patient presented with limited opening of the mouth due to trismus. These factors prevented further procedures and a second visit was scheduled.

**Figure 4:**
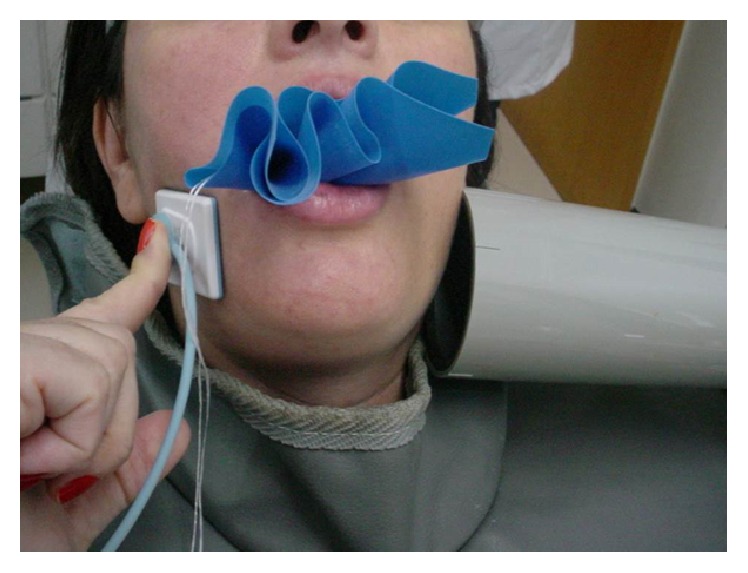
Extraoral technique used for the mandibular molar during root canal therapy.

**Figure 5:**
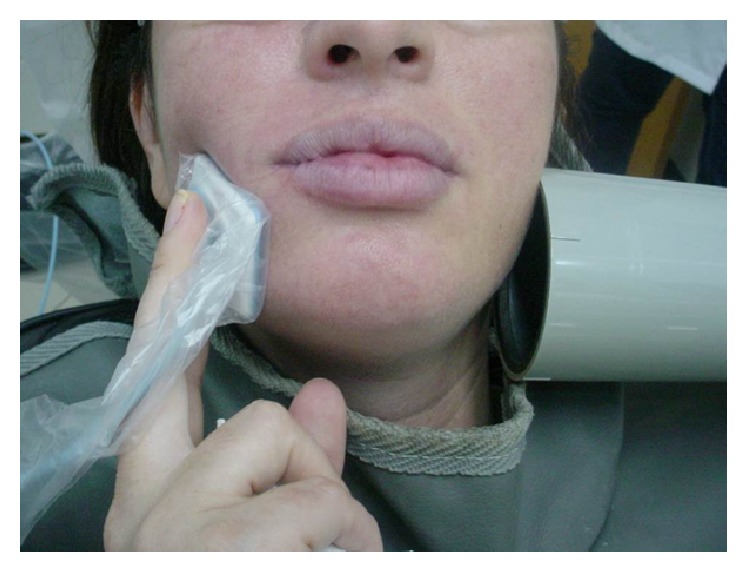
Extraoral technique used for final radiograph after root canal therapy.

**Figure 6:**
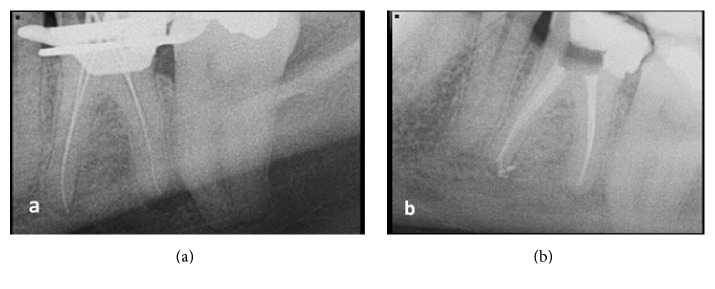
(a) Radiograph for working length confirmation: case report number 2. (b) Final radiograph: case report number 2.

A bite block for opening the mouth was deemed necessary during the second visit in order to achieve a better field of vision and make the patient comfortable. Subsequent to the rubber dam placement and irrigation procedures, WL determination was performed using an EAL. For greater accuracy of the WL estimation, a periapical radiograph was necessary; nevertheless, the limited mouth opening and the gag reflex were obstacles for the use of an intraoral sensor (Schick Fona Elite) (Figure 4). The extraoral technique was then performed. The patient placed the sensor on the face parallel to the tooth, and the X-ray cone was placed with a horizontal angulation of −35° and perpendicular to the sensor. The canals were then instrumented using Easy Logic 25.06 NiTi files (Easy) under irrigation with 5.25% NaOCl and 17% EDTA. The canals were filled with gutta-percha points and the sealer AH plus (Dentsply De Trey, Konstanz, Germany) using a warm vertical compaction. Finally, a temporary restoration was placed. Postoperative radiography was performed using the extraoral technique (Figures 5 and 6) with 0.5 s as the exposition time.

## 4. Discussion

The gag reflex is a physiological protection mechanism triggered by the contact of foreign bodies with the pharynx, larynx, or trachea [1] or a manifestation of an anxiety state [9]. However, when this mechanism is exaggerated, it can be undesirable and prevent the healthcare provider from providing good results [10]. In both the cases, the severe gag reflex made it impossible for the endodontist to acquire intraoral periapical radiographs, which are usually obtained in a routine procedure. Besides, the phobic behavior of patient number 1 and the trismus in patient number 2 made it even more difficult to achieve good quality images.

Different strategies, including intravenous sedation [2], behavioral approaches [11], distraction methods, acupuncture [12], nitrous oxide sedation, and comportment therapy [10], may be used to diminish the gag reflex.

Friedman and Weintraub [13] have shown that clinical procedures such as placing table salt for 5 s on the anterior part of the tongue may eliminate gagging in some patients, thus permitting radiographs and impression procedures. This technique would eliminate the gag reflex due to simultaneous stimulation of the nerve of the tympanic cord and the gustatory papillae of the anterior two-thirds of the tongue. However, this technique was not as effective as the results achieved by sedation using nitrous oxide [1].

The exaggerated gag reflex in patients may present a serious obstacle in achieving good quality images, which are important for RCT. This reflex might be minimized by using high-speed films, placing the films in cold water, and mouth washing with cold water before the placement of the film. In addition a smaller sensor or film might be helpful in some cases. Using medication in conjunction with topical anesthetics may lead to proper results when the reflex is mild to moderate; however, in exaggerated cases, the effect might be contrary because the numb sensation in the palate or pharynx may be sufficient to trigger the reflex [10].

If none of the aforementioned techniques provides satisfactory results, the extraoral technique can be used as an alternative to achieve good quality periapical radiographs. Several studies, such as those by Newman et al. [6], Chen et al. [14], Saberi et al. [7], and Kumar et al. [15], have demonstrated the efficacy of the extraoral technique and its variations for both the upper and lower molars. This technique does not aim to replace the traditional intraoral technique; however, it can be used for specific cases of patients presenting with severe gag reflex and/or with limitation of opening the mouth [6, 7, 14]. Likewise, it could also be recommended for patients with mental illness, pediatric patients, and patients with phobia [4, 6].

This technique has some disadvantages. It cannot be used for anterior teeth, and the quality of images acquired using this technique might not be as sharp as those acquired using the conventional intraoral technique [6, 7, 14] because the tooth-film distance in this technique is greater than that in the conventional technique [14]. Moreover, the exposition time in this technique is higher than that in the intraoral technique; however, this factor can be minimized by reduction in the number of failed intraoral radiographs [7].

In both the cases, the same radiography device was used (Spectro II 70X, Dabi-Atlante, Ribeirão Preto, Brazil) with 70 kVp e 8 mA. In the first case, when a conventional film (Kodak E-Speed) was used, the exposition time was set at 1.5 s, while, in the second case, with further utilization of a digital sensor, the exposition time was reduced to 0.5 s. This procedure is in accordance with the principle of as low as reasonably achievable (ALARA), which recommends using the minimum dose of radiation necessary to achieve an accurate image [16]. An alternative to this technique should be the use of electronic apex locator (EAL), as it has been showed as a reliable technique for working length determination when combined with radiographs [3]. With recent use of cone-beam computed tomography (CBCT), the presence of a previous examination should be considered for WL determination when an examination is already available. A recent in vitro study has shown that CBCT is reliable for this step; however, there is no indication to use CBCT exclusively for WL determination [17].

## 5. Conclusion

Extraoral periapical radiographic technique was successfully used for the upper and lower molars in the 2 reported patients in whom the conventional intraoral radiographic technique could not be used due to severe gag reflex, trismus, and phobic behavior.
